# Effect of Distal Fragment Length on Construct Stability in an Extra-articular Distal Tibial Fracture Model Fixed With Locked Intramedullary Nailing: A Biomechanical Study

**DOI:** 10.7759/cureus.83355

**Published:** 2025-05-02

**Authors:** Nitin Chauhan, Sushil Kumar, Tarkik Thami, Navin Kumar, Sharad Prabhakar, Mandeep Dhillon, Siddhartha Sharma

**Affiliations:** 1 Orthopaedics, Postgraduate Institute of Medical Education and Research, Chandigarh, Chandigarh, IND; 2 Mechanical Engineering, Indian Institute of Technology (IIT) Ropar, Ropar, IND

**Keywords:** biomechanical study, distal tibia fracture, expert tibia nailing, extra-articular distal tibial fracture, extra-articular fracture, interlocking nail tibia, saw bone

## Abstract

Introduction

Fractures of the distal tibia are complex injuries with high complication rates, which include delayed union, non-union, and wound complications like dehiscence and infection. The two commonly employed definite internal fixation modalities include locked intramedullary (IM) nailing and plating. There is controversy regarding the superiority of the fixation construct, although nailing is proven to be more biological and devoid of soft tissue complications. There is also no consensus regarding the minimum distance of the fracture from the tibial plafond that is amenable to nailing of the fracture. Hence, the present study is designed to evaluate the effect of distal fragment length relative to the total length of the tibia, which makes it stable enough for IM nailing to be effective.

Methods

A prospective biomechanical study was performed using 28 fourth-generation composite tibial sawbones. Osteotomies were created at 12%, 15%, 20%, and 25% of the total tibial length (38 cm) from the distal articular surface, forming four experimental groups (A-D, n=7 each). All models were stabilized with 10 mm stainless-steel interlocking nails. Mechanical testing was conducted using a servo-hydraulic fatigue testing machine and included mediolateral (ML) and anteroposterior (AP) three-point bending, as well as cyclic axial loading. Outcome measures included bending stiffness, construct laxity (neutral zone), fracture gap angle, axial micromotion, and construct failure.

Results

The bending stiffness of all constructs tended to be lower in the AP plane than in the ML plane. The neutral zone of all groups tended to be higher in the AP plane than in the ML plane. The peak fracture gap angle tended to be higher in the AP plane than in the ML plane. Group A (shortest distal fragment length) demonstrated significantly lower AP stiffness, higher AP neutral zone, and higher AP peak fracture gap angle as compared to group D (longest distal fragment length). Group A demonstrated significantly greater instability in the AP plane than Group D. No statistically significant difference was found in the stability parameters on medio-lateral three-point bending and axial compressive testing.

Conclusion

The results of this biomechanical study show that comminuted extra-articular distal tibial fractures show significant instability in the sagittal plane when the length of the distal fragment is 12% of the total tibial length.

## Introduction

Fractures of the distal tibia account for 0.7% of all fractures and less than 10% of lower limb fractures. These are more prevalent worldwide in adolescent boys aged 10-20 years [1-4]. These are complex injuries with high complication rates, that include delayed union, non-union, and wound complications like dehiscence and infection due to anatomical and biomechanical reasons like subcutaneous position, surrounded by only tendons, leading to poor vascular supply and a metaphyseal wide area of osseous tissues [5-11]. 

Conservative treatment is generally limited to distal tibial fractures that are both stable and non-displaced, or in cases where surgical intervention poses substantial risk due to patient comorbidities. Conversely, most displaced distal tibial fractures in adults are best managed surgically to ensure proper alignment, stability, and functional recovery [6]. The two most commonly used definitive internal fixation methods are locked intramedullary (IM) nailing and plate fixation [12-14]. Conventional plating techniques employ the principles of open reduction under direct visualization and absolute fracture stability [5,15,16]. However, it has been linked to significant soft tissue disruption and periosteal damage, which are known contributors to postoperative wound issues and infection [5-7]. 

The biomechanical limitations of early IM nail designs, specifically the scarcity of distal locking options, rendered them less favorable for stabilizing distal tibial fractures, where secure metaphyseal fixation is critical [17-19]. To address this limitation, nail designs were modified to include additional distal locking options. This was accomplished by incorporating extra distal holes, allowing placement of an additional locking screw to enhance fixation stability [19-21]. This innovation led to the development of modern IM nails, which now feature three to four distal locking options. These locking holes are strategically oriented in the anteroposterior (AP) and oblique planes, offering improved fixation and enhanced biomechanical stability in the distal segment [19-26].

It is interesting to note that although there have been several advances in nail design, the problem of malunion persists. The impact of distal fragment length on the overall stability of IM nail constructs has not been thoroughly explored. Since stability is directly proportional to the working length of an IM nail, decreased working length would reduce the stability of the construct. Also, to our knowledge, no studies have evaluated the biomechanics or stability of distal tibial fractures treated with IM nailing, nor have any investigated the correlation between these factors and the likelihood of malunion or clinical outcomes. Hence, the present study is designed to evaluate the effect of distal fragment length relative to the total length of the tibia, which makes it stable enough for IM nailing to be effective. The purpose of this study is to explore how distal fragment length affects fracture stability in a distal tibial extra-articular gap fracture model stabilized by locked IM nailing, and to determine the shortest possible length of the distal fragment of an unstable extra-articular distal tibial fracture that can be effectively stabilized by a locked IM nail.

## Materials and methods

Study framework

This prospective biomechanical study utilized fourth-generation composite tibial sawbones, with no involvement of living human subjects, cadaveric specimens, or animal models. The study was designed exclusively with synthetic bone models to simulate the mechanical properties of the tibia. Although biological specimens were not used, ethical approval for the study was obtained from the Institutional Ethics Committee of the Postgraduate Institute of Medical Education and Research (PGIMER), Chandigarh, India (approval number: IEC-11/2017-747). The research was conducted by PGIMER in collaboration with the Indian Institute of Technology, Ropar, from January to December, 2018.

Sample organization and grouping

A total of 28 synthetic tibial bone models (Sawbones, 4th Generation, ERP #3402; Pacific Research Laboratories, Inc., Vashon, Washington, United States) were used, each measuring 38 cm in length with a 10 mm medullary canal diameter. These models replicate the mechanical properties of human bone and have been validated for mechanical testing in prior studies [21].

**Figure 1:**
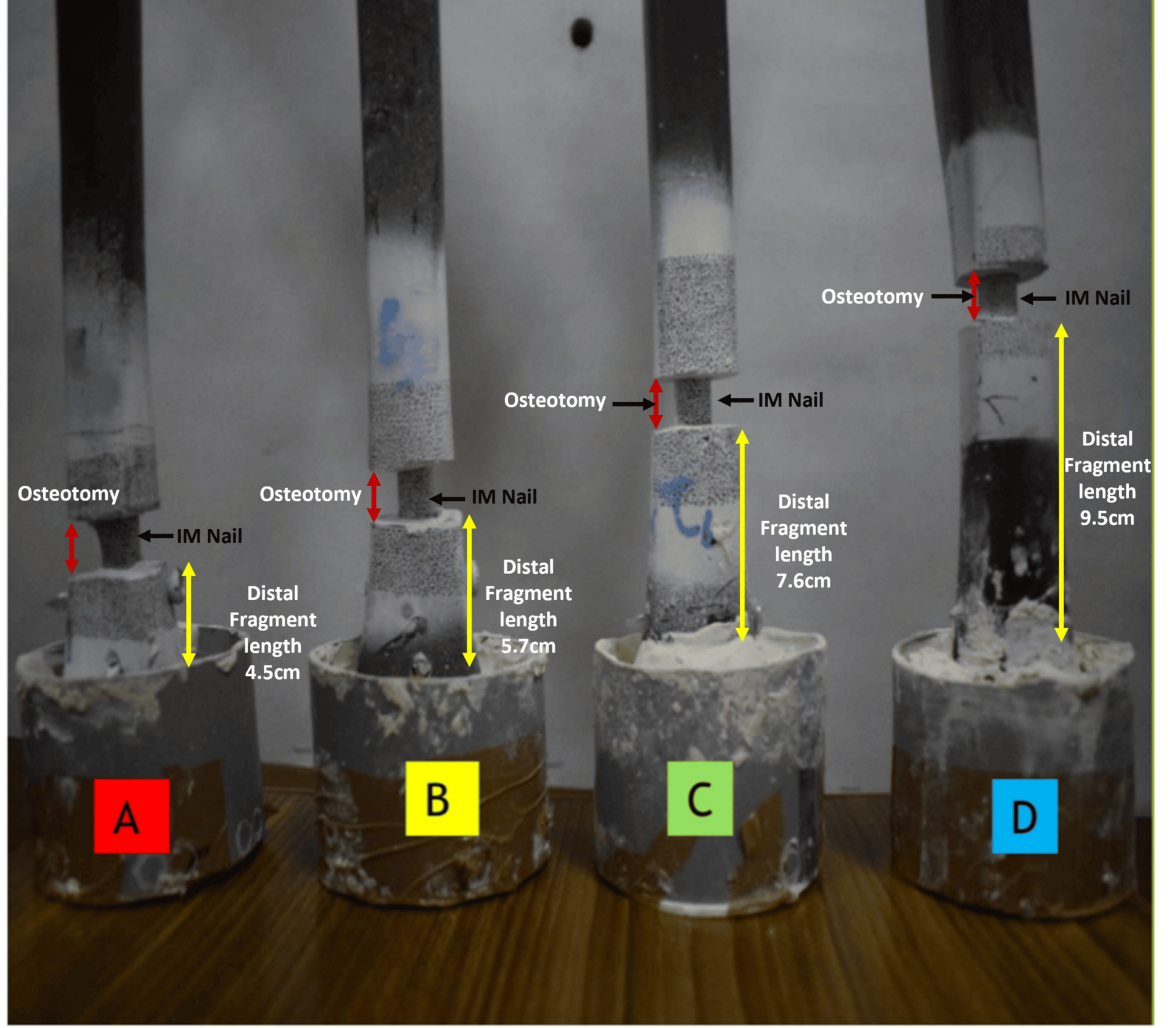
Group classification and distal potting Visual representation of four experimental groups (A–D) based on distal fragment length post-osteotomy. Each construct (N = 7 per group) was fixed with a locked intramedullary nail, with distal ends embedded in custom jigs using Bondo® lightweight body filler (3M Company, Maplewood, Minnesota, United States). IM: intramedullary

Based on established biomechanical protocols and prior studies [21-24], a sample size of seven specimens per group was selected to ensure adequate statistical power while accommodating practical limitations. The specimens were divided into four experimental groups (A-D), each simulating comminuted extra-articular distal tibial fractures with varying distal fragment lengths, as follows: Group A: 12% of the total tibial length (4.5 cm), Group B: 15% of the total tibial length (5.7 cm), Group C: 20% of the total tibial length (7.6 cm), Group D: 25% of the total tibial length (9.5 cm) (Figure 1).

Groups A and B were designed to simulate extra-articular distal tibial fractures (Arbeitsgemeinschaft für Osteosynthesefragen/Orthopedic Trauma Association (AO/OTA) 43C3), while Groups C and D represented distal third diaphyseal fractures (AO/OTA 42C3).

Osteotomy protocol and implant stabilization

To simulate comminuted extra-articular distal tibial fractures, a transverse osteotomy measuring 1 cm was carefully created at designated levels from the distal articular surface, as per group classification (Figure 1). The osteotomy was performed after nail placement to ensure the construct remained intact during the process. No contact was maintained between the fracture ends, reflecting an unstable, gap-type fracture without fibular support.

All tibial models were stabilized using 10 mm × 38 cm stainless steel interlocking IM nails (Siora Surgicals Pvt. Ltd., Sonipat, Haryana, India). Prior to insertion, each canal was reamed to 11 mm to accommodate the nail. To ensure consistency across specimens, the distal end of the nail was located within 5 mm of the tibial plafond.

Each construct was secured using two proximal locking screws (one static and one dynamic). Additionally, three distal screws were placed: two in the mediolateral (ML) plane and one in the AP plane. All screws achieved bicortical fixation. Proximal locking was performed using a guided jig provided by the manufacturer, while distal locking was achieved under fluoroscopic guidance using the perfect circle technique to ensure precise screw placement.

Mechanical testing methodology

**Figure 2:**
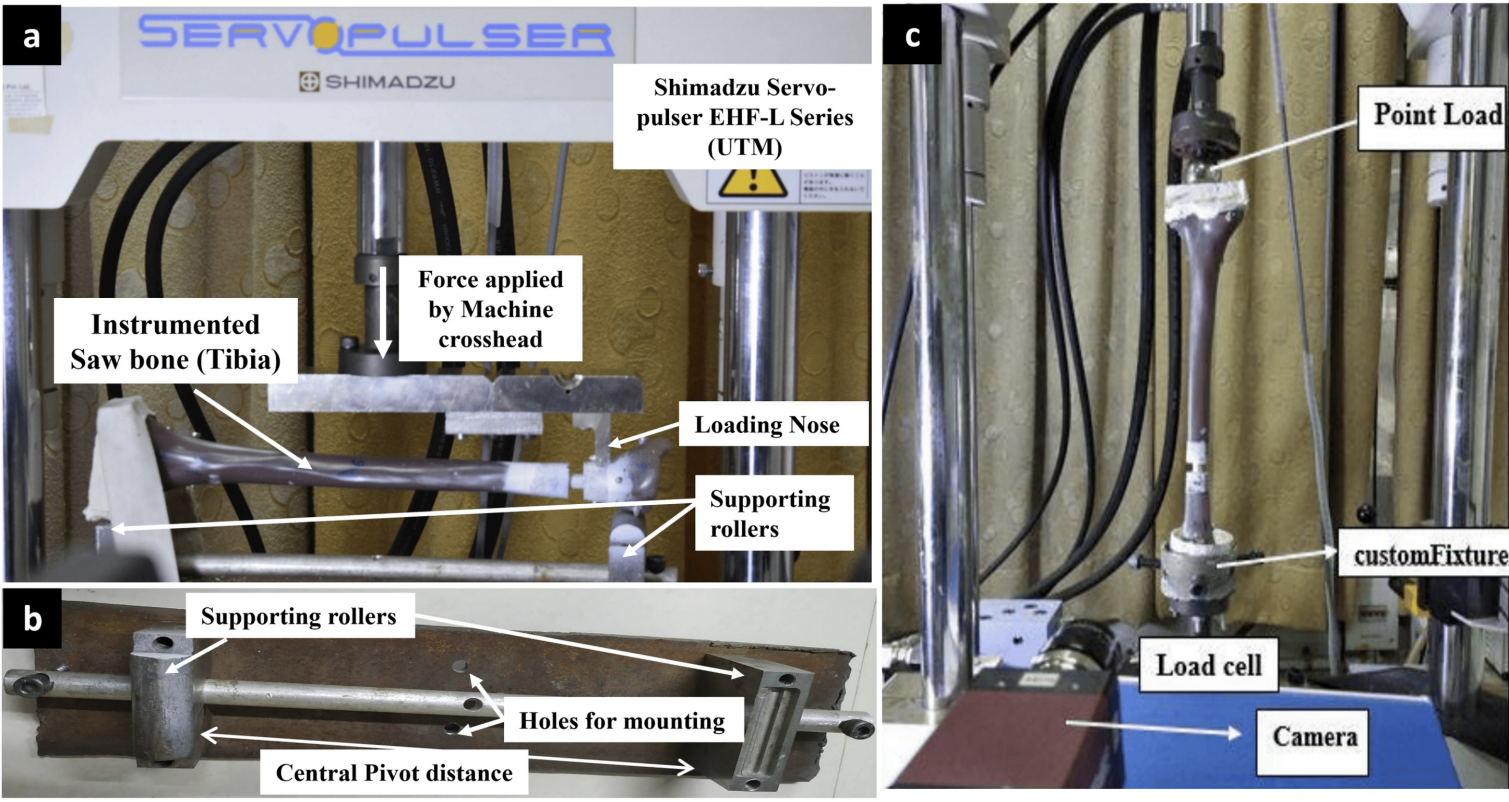
(a) Test setup showing instrumented tibia positioned for anteroposterior and mediolateral bending tests; (b) Custom-made fixation used for securing the instrumented tibia; (c) Test setup for cyclic axial compression testing using sinusoidal axial loading UTM: universal testing machine Manufacturer details: Shimadzu Servopulser EHF-E Series, Shimadzu Corporation, Kyoto, Japan

**Figure 3:**
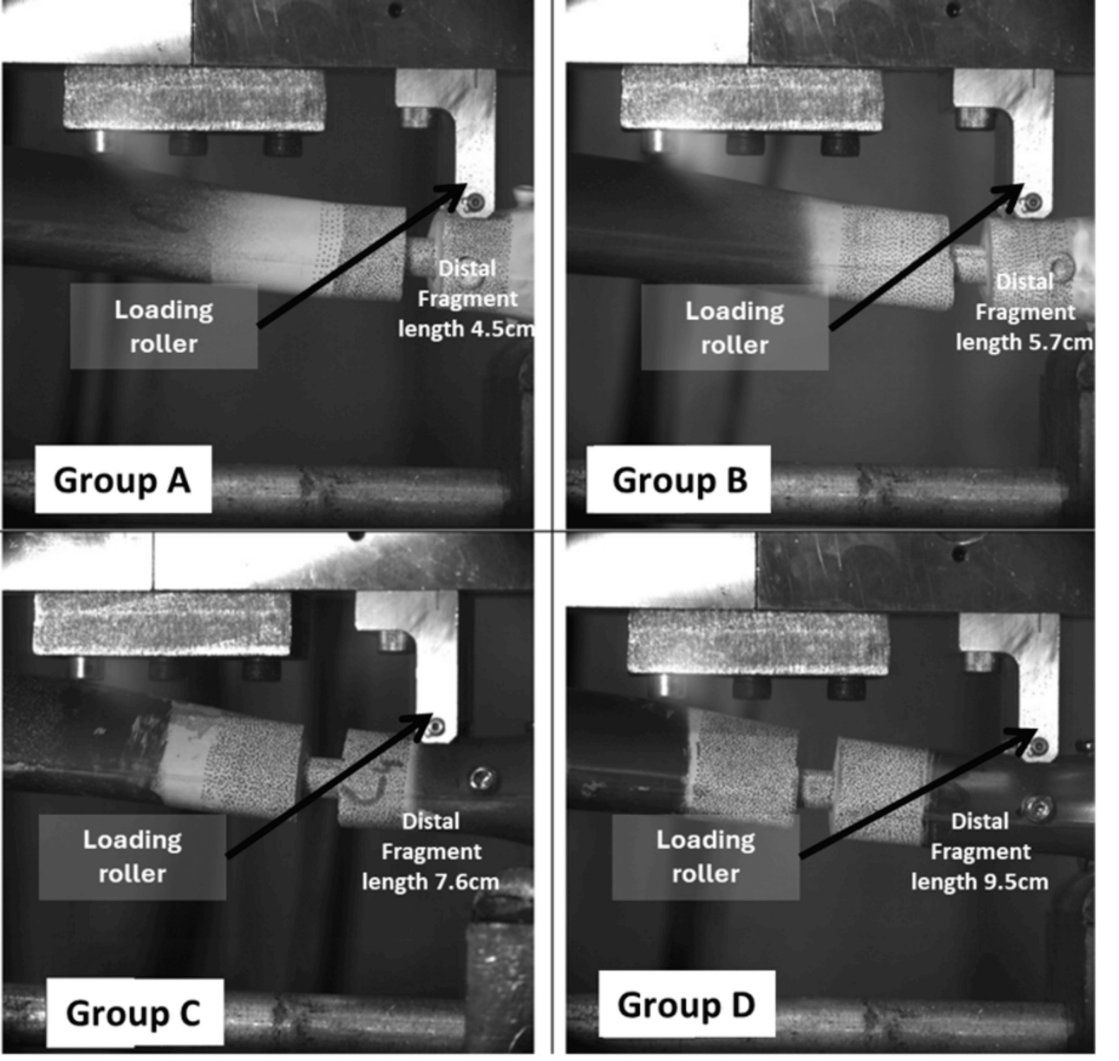
Representative images showing the offset three-point mediolateral bending test setup for Groups A, B, C, and D, respectively. Each construct is placed on two support rollers with a loading roller applying an offset load to simulate three-point bending in the coronal (mediolateral) plane.

Based on previous studies [21-24], a servo-hydraulic testing machine (Shimadzu Servopulser EHF-E Series Fatigue Testing Unit; Shimadzu Corporation, Kyoto, Japan) was used to perform the mechanical tests, which included ML bending in the elastic range, AL bending in the elastic range, and sinusoidal axial loading (Figure 2). Figure 3 demonstrates the offset three-point ML bending test setup used for Groups A through D, with each construct positioned on two support rollers and an offset load applied via a loading roller to simulate bending in the coronal (ML) plane.

ML and AP bending testing was done by the three-point bending test. A custom-built jig, incorporating two adjustable support rollers, was developed for this purpose (Figure 2b). The elastic range of the test specimen was assessed through initial trial evaluations. A load of about 500 N, within the elastic limit, was applied at an actuator speed of 0.05 mm/second to the implanted tibia, followed by unloading at a reduced speed of 0.0025 mm/second. The load and displacement data were recorded, and the mean slope was computed between 50 and 500 N, which corresponds to the linear elastic region.

As described in previous studies [21,23], axial compression testing was conducted by potting the proximal and distal ends of the test specimens in custom-made jigs using Bondo® lightweight body filler (3M Company, Maplewood, Minnesota, United States) (Figure 2c). At the proximal tibia, the axial force was applied 10 mm medially and 10 mm posteriorly to the medial intercondylar tubercle, while the distal load was directed to the center of the inferior articular surface. A cyclical loading protocol (16000 cycles at 75 Hz), which has been validated in previous studies, was followed, and 4000 cycles were done [21,22]. Specimens were subjected to loads ranging from 80 to 800 N, maintaining a 10:1 load ratio. The 80 N load corresponds to the body weight of an individual weighing 82 kg. Micromotion at the fracture site and strain distribution were studied through high-speed cameras (Vic-3D microsystem; Correlated Solutions Inc., Columbia, South Carolina, United States) and digital image correlation software. Bending stiffness, construct laxity, fracture gap angle, axial micromotion, and construct failure were noted as outcomes after the nailing procedure (Table 1).

**Table 1:** Biomechanical outcome measures used in the study Summary of biomechanical outcome measures evaluated, including definitions, test methods, statistical analyses, and clinical relevance. Bending stiffness, neutral zone, and fracture gap angle were assessed in both anteroposterior (AP) and mediolateral (ML) planes using three-point bending. Axial micromotion was recorded under cyclic loading.

Outcome Measure	Definition	Plane/Test Type	Units / Format	Statistical Analysis	Clinical Relevance	
Bending Stiffness	Resistance of the construct to bending, calculated as the slope of the load-displacement curve within the linear elastic range.	Anteroposterior (AP) and Mediolateral (ML); Three-point bending	Nm/degree; Mean ± SD	Paired t-test for AP vs ML; ANOVA with Tukey HSD for between-group comparison	Indicates overall rigidity of the construct in resisting bending loads.	
Neutral Zone (Laxity)	Range of displacement with minimal resistance before linear stiffness begins. Indicates construct laxity.	AP and ML; derived from hysteresis loop during bending	mm; Mean ± SD	Paired t-test for AP vs ML; ANOVA with Tukey HSD	Higher values suggest reduced stability and potential for early micromotion.	
Fracture Gap Angle	Angular deformation at the fracture site under loading. Reflects the degree of motion at the osteotomy site.	AP and ML; determined from angular change at fracture site during bending	Degrees (°); Mean ± SD	Paired t-test for AP vs ML; ANOVA with Tukey HSD	Higher angles imply poor fixation and risk of malalignment or delayed union.	
Axial Micromotion	Displacement at the fracture site during cyclic axial compression, assessed at 40, 2000, and 4000 cycles.	Axial compression; cyclic loading test	mm; Mean ± SD	ANOVA across groups (no post-hoc needed)	Excessive micromotion may compromise healing under cyclic physiological loading.	
Construct Failure	Occurrence of any structural failure such as screw loosening, nail deformation, or sawbone fracture during testing.	Observed post-test in all directions	Yes/No (Descriptive)	Descriptive reporting only	Important for understanding construct integrity and practical implant performance.	

Outcome measures

**Figure 4:**
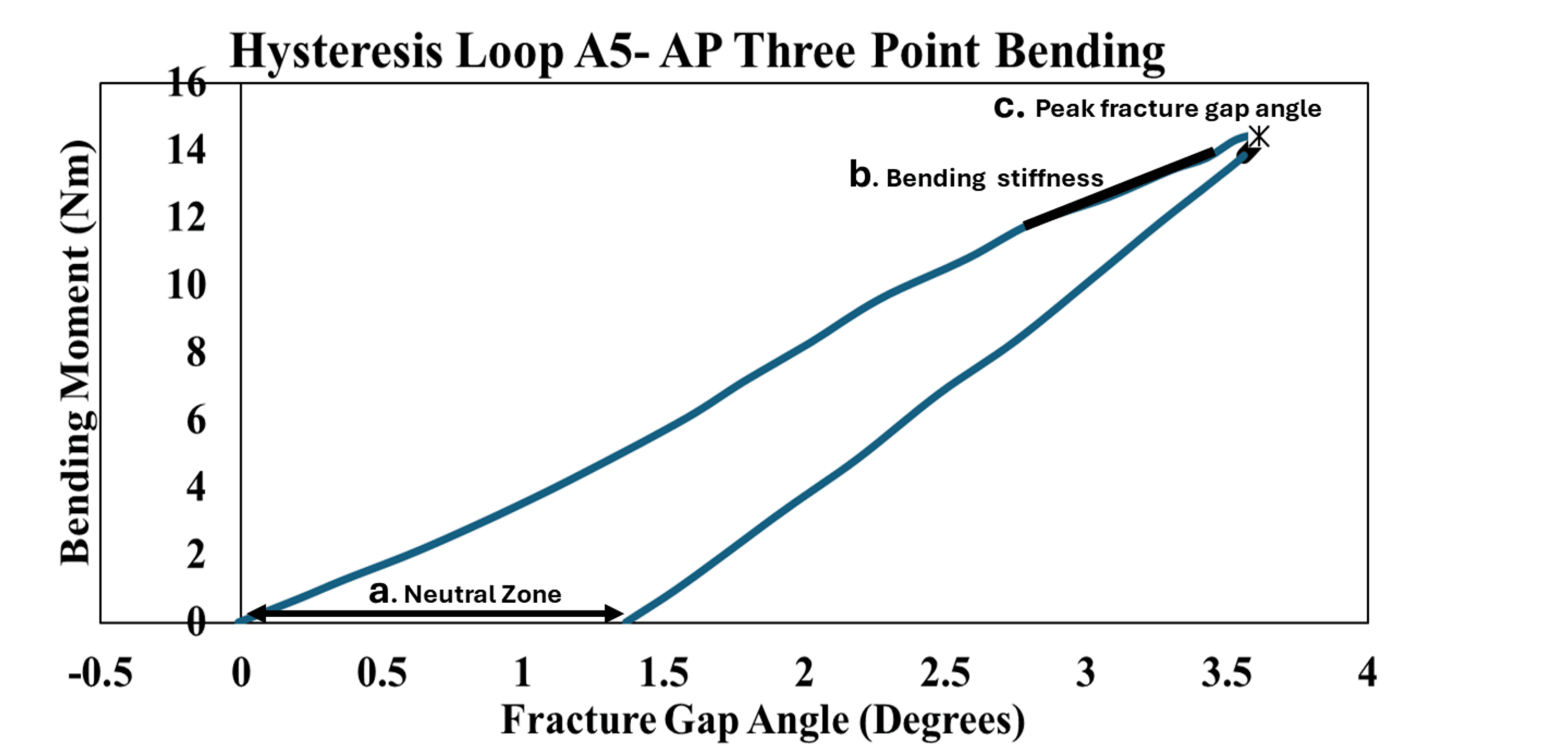
Hysteresis loop for anteroposterior bending – construct A5 The hysteresis loop displays the mechanical response of sample A5 during anterior-posterior three-point bending testing, with bending moment (N·m) plotted against fracture gap angle (degrees). The graph reveals three critical parameters: (a) the Neutral Zone, representing the initial low-resistance phase of deformation; (b) the linear region used to calculate bending stiffness from the slope of the curve; and (c) the peak fracture gap angle indicating maximum deformation capacity. The enclosed area of the loop quantifies energy dissipation during loading cycles, while "Mean ± SD" indicates averaged data.

**Figure 5:**
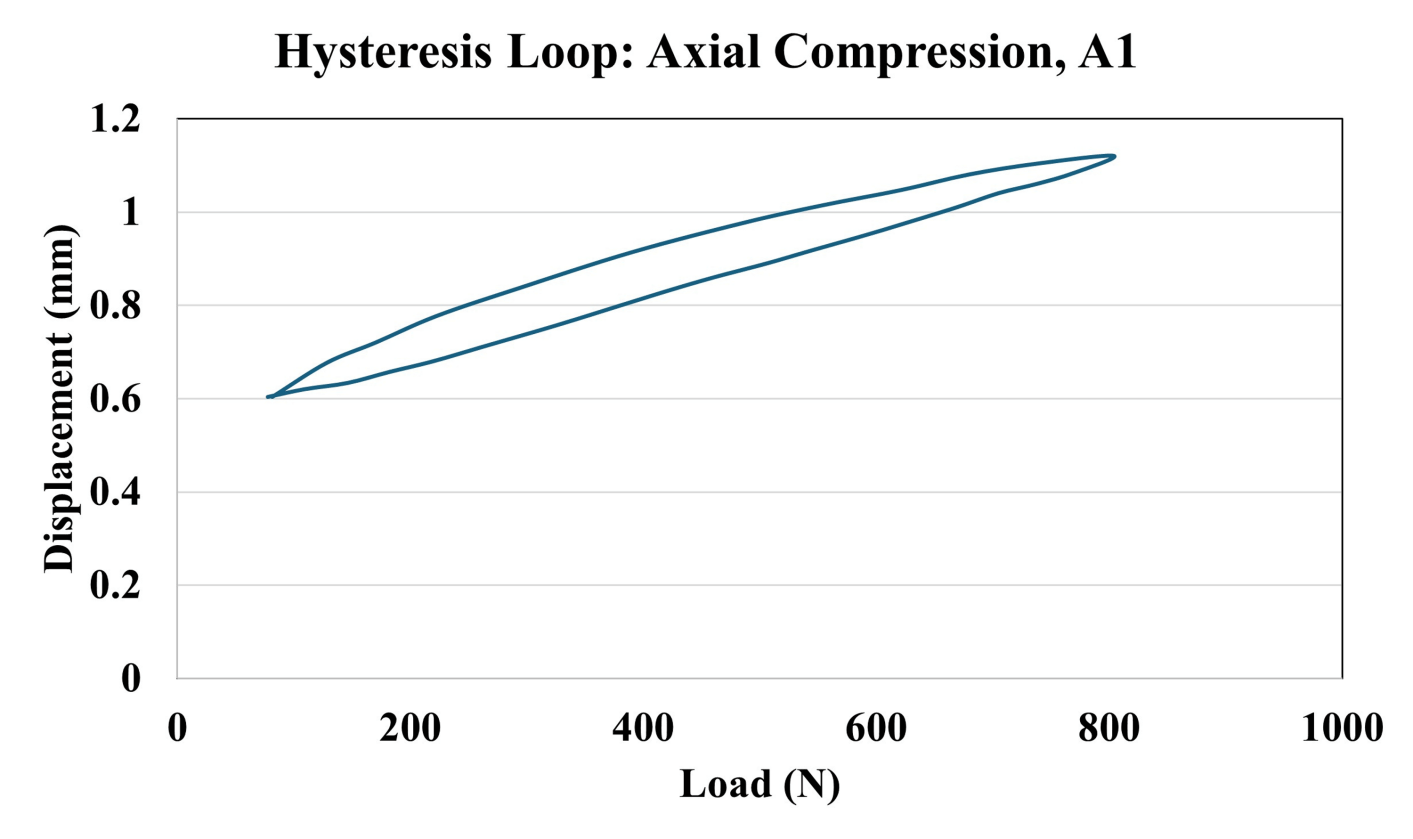
Hysteresis loop for cyclic axial compression, A1 construct This graph illustrates the load-displacement relationship of construct A1 during cyclic axial loading. Data were recorded at 40, 2000, and 4000 cycles to measure micromotion and stiffness. The hysteresis loop reflects how the construct responded to repeated loading and unloading. The width of the loop indicates energy loss due to internal friction and motion within the construct. The slope of the curve represents axial stiffness — a steeper slope indicates a stiffer, more stable construct. A smaller displacement and narrower loop over time suggest better stability and less micromotion, which are important for successful fracture healing.

Table 1 shows the measurements used to check how well each construct performed. Bending stiffness represents how much the construct resists bending and was measured in both AP and ML directions. It was calculated by dividing the applied force by the resulting angle at the fracture site. As shown in the representative hysteresis loop, key regions used for analysis: (a) the neutral zone, (b) the linear region used for stiffness calculation, and (c) the peak fracture gap angle (Figure 4). The neutral zone refers to the small range of movement where the construct moves easily with minimal force; a smaller neutral zone indicates better stability. The fracture gap angle is the angle observed at the fracture site when the construct is loaded from above; smaller angles suggest improved bone-holding capacity. Axial micromotion, shown during cyclic compression at 40, 2000, and 4000 cycles, measures vertical movement (Figure 5). Axial stiffness was calculated using the formula 720 divided by the difference between peak and minimum displacement values. Construct failure was recorded if any screw loosening, breakage, or sawbone damage occurred by the end of testing.

Data analysis

Data from the ML bending test, AP bending test, and axial compression test were compiled using Microsoft Excel for Macintosh (Microsoft, Redmond, Washington, United States) and analyzed with SPSS Statistics for Macintosh, Version 17.0 (Released 2008; SPSS Inc., Chicago, Illinois, United States). The Shapiro-Wilk test was used to assess the normality of the data. Categorical variables were compared using the chi-square test. For quantitative variables, the Wilcoxon rank-sum test was applied to non-parametric data, while parametric data were analyzed using one-way ANOVA, followed by Tukey’s HSD (honestly significant difference) post-hoc test for pairwise comparisons. A p-value of < 0.05 was considered statistically significant. Continuous variables were categorized where appropriate to simplify statistical interpretation.

## Results

Analysis of three-point bending

Three-point bending tests were performed within the elastic range in both the ML (coronal) and AP (sagittal) planes. The Shapiro-Wilk test identified normal distribution of data for the parameters of AP and ML bending stiffness, AP fracture gap angle, and AP neutral zone. The other parameters (ML gap angle and ML neutral zone) were found to have a non-normal distribution.

Bending stiffness

**Figure 6:**
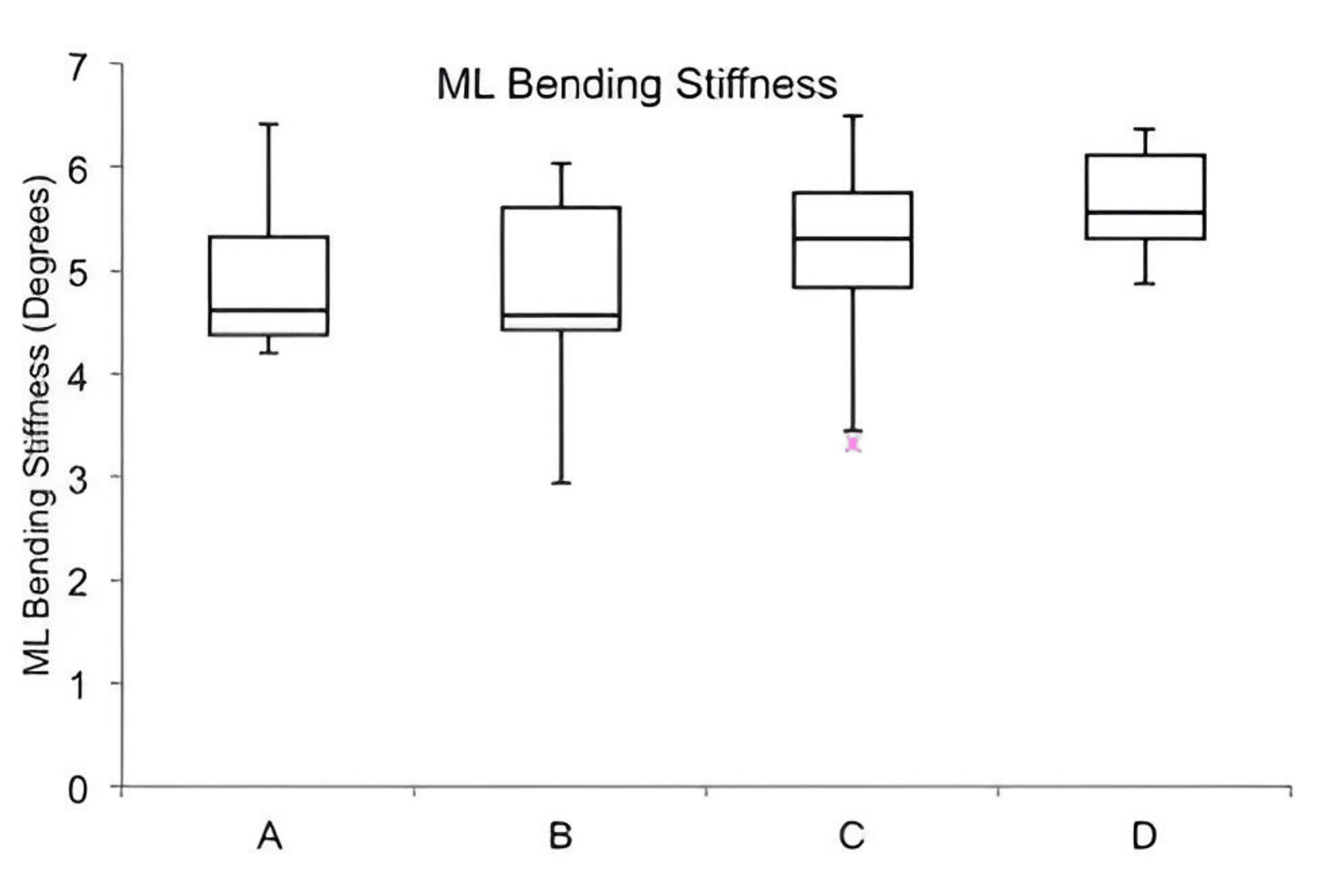
Comparison of mediolateral bending stiffness Box plot illustrating mediolateral bending stiffness in Groups A–D. Median is marked by the line inside the box; box edges show the interquartile range. Whiskers represent the range. N = 7 constructs per group. Group D showed the highest median stiffness, indicating better resistance to side-to-side bending.

In biomechanical testing, constructs exhibited higher bending stiffness in the ML plane compared to the AP plane, with a statistically significant difference observed only in Group A (p = 0.01) (Table 2). Group D showed the highest stiffness in both planes, while Group A had the lowest AP stiffness. ML stiffness varied across groups, with the lowest in Group B and the highest in Group D, though these differences were not statistically significant (Figure 6). In contrast, AP bending stiffness increased progressively from Group A to D, with post-hoc analysis confirming a significant difference between Group A and Group D (p = 0.021) (Table 3), while other group comparisons were not significant.

**Table 2:** Comparison of anteroposterior and mediolateral bending stiffness Data represented as Mean ± Standard Deviation (SD). Comparison performed using paired t-test between AP and ML stiffness within each group. A p-value < 0.05 was considered statistically significant. Each group included 7 specimens (N = 7).

Group	AP Stiffness (Nm/deg), mean ± SD	ML Stiffness (Nm/deg), Mean ± SD	Statistical Test	Test Statistic (t)	p-value
A	4.15 ± 0.36	4.95 ± 0.80	Paired t-test	3.01	0.01
B	4.63 ± 0.82	4.80 ± 1.05	Paired t-test	0.91	0.38
C	4.69 ± 0.50	5.18 ± 1.03	Paired t-test	1.69	0.13
D	5.16 ± 0.62	5.66 ± 0.56	Paired t-test	2.12	0.06

**Table 3:** Post-hoc Tukey HSD analysis for anteroposterior stiffness Tukey’s HSD post-hoc test following one-way ANOVA to compare anteroposterior stiffness across groups. p-value < 0.05 was considered significant. F-statistic for ANOVA = 4.78. N = 7 per group. HSD: honestly significant difference

Comparison	Tukey Q Statistic	p-value
A vs B	2.14	0.45
A vs C	2.36	0.36
A vs D	4.43	0.021
B vs C	0.22	0.9
B vs D	2.29	0.39
C vs D	2.07	0.47

Peak fracture gap angle

Constructs consistently exhibited higher peak fracture gap angles in the AP plane compared to the ML plane, with significant differences observed in Group A (p = 0.01) and Group D (p = 0.04) (Table 4). The AP angle progressively decreased from Group A to Group D, indicating improved sagittal plane stability with increasing distal fragment length. This trend was statistically confirmed by one-way ANOVA and Tukey’s post-hoc test, which showed a significant difference between Group A and Group D (p = 0.01), while other comparisons were not significant (Table 5). Although the ML angle was highest in Group B and lowest in Group D, no significant intergroup differences were found in the ML plane.

**Table 4:** Comparison of anterioposterior vs mediolateral peak fracture gap angle Paired t-test used to compare AP and ML fracture gap angles within each group. A p-value < 0.05 was considered significant. N = 7 per group.

Group	AP Angle (deg), Mean ± SD	ML Angle (deg), Mean ± SD	Statistical Test	Test Statistic (t)	p-value
A	3.50 ± 0.27	3.00 ± 0.46	Paired t-test	3.21	0.01
B	3.11 ± 0.53	3.09 ± 0.83	Paired t-test	0.14	0.48
C	3.09 ± 0.32	2.85 ± 0.59	Paired t-test	1.45	0.18
D	2.82 ± 0.32	2.55 ± 0.22	Paired t-test	2.49	0.04

**Table 5:** Post-hoc analysis of peak anteroposterior fracture gap angle Tukey’s HSD post-hoc analysis of peak anteroposterior fracture gap angles among study groups. Tukey HSD Q statistics and p-values are presented. One-way ANOVA followed by Tukey's HSD was used. F-statistic = 5.12. Significance set at p < 0.05. HSD: honestly significant difference

Treatments pair	Tukey HSD Q statistic	Tukey HSD p-value
A vs B	2.8039	0.22
A vs C	2.9359	0.19
A vs D	4.856	0.01
B vs C	0.1321	0.9
B vs D	2.0521	0.48
C vs D	1.92	0.53

Neutral zone

Neutral zone comparison revealed that constructs generally exhibited a significantly larger neutral zone in the AP plane compared to the ML plane. Within-group comparisons showed the AP neutral zone was higher on average, with a statistically significant difference noted only in Group A (Table 6). The ML neutral zone was lowest in Group D and highest in Group B, though differences across groups were not statistically significant on one-way ANOVA. In contrast, the AP neutral zone demonstrated a progressive decrease from Group A to Group D, with significant differences observed between groups A and C and A and D, as confirmed by post-hoc analysis (Table 7). These results highlight directional variability in construct stability, particularly in the AP plane.

**Table 6:** Comparison of neutral zone (anteroposterior vs. mediolateral ) Paired t-test used to compare anteroposterior and mediolateral neutral zones. N = 7 per group. p < 0.05 was considered statistically significant.

Group	AP Neutral Zone (mm), mean ± SD	ML Neutral Zone (mm), mean ± SD	p-value	Test Statistic (t)
Overall (N = 28)	0.72 ± 0.36	0.58 ± 0.37	0.02	t = 2.32
A	1.09 ± 0.31	0.57 ± 0.30	0.004	t = 4.16
B	0.75 ± 0.41	0.69 ± 0.47	0.41	t = 0.85
C	0.58 ± 0.21	0.45 ± 0.44	0.33	t = 1.06
D	0.44 ± 0.15	0.35 ± 0.22	0.19	t = 1.31

**Table 7:** Post-hoc analysis of anteroposterior neutral zone (one-way ANOVA followed by Tukey HSD) ANOVA F = 6.23. p < 0.05 was considered significant. Each group included N = 7 constructs. HSD: honestly significant difference

Treatment Pair	Tukey HSD Q-statistic	p-value
A vs B	3.0636	0.16
A vs C	4.6095	0.016
A vs D	5.8490	0.002
B vs C	1.5460	0.68
B vs D	2.7855	0.23
C vs D	1.2395	0.80

Analysis of cyclic compressive loading

**Figure 7:**
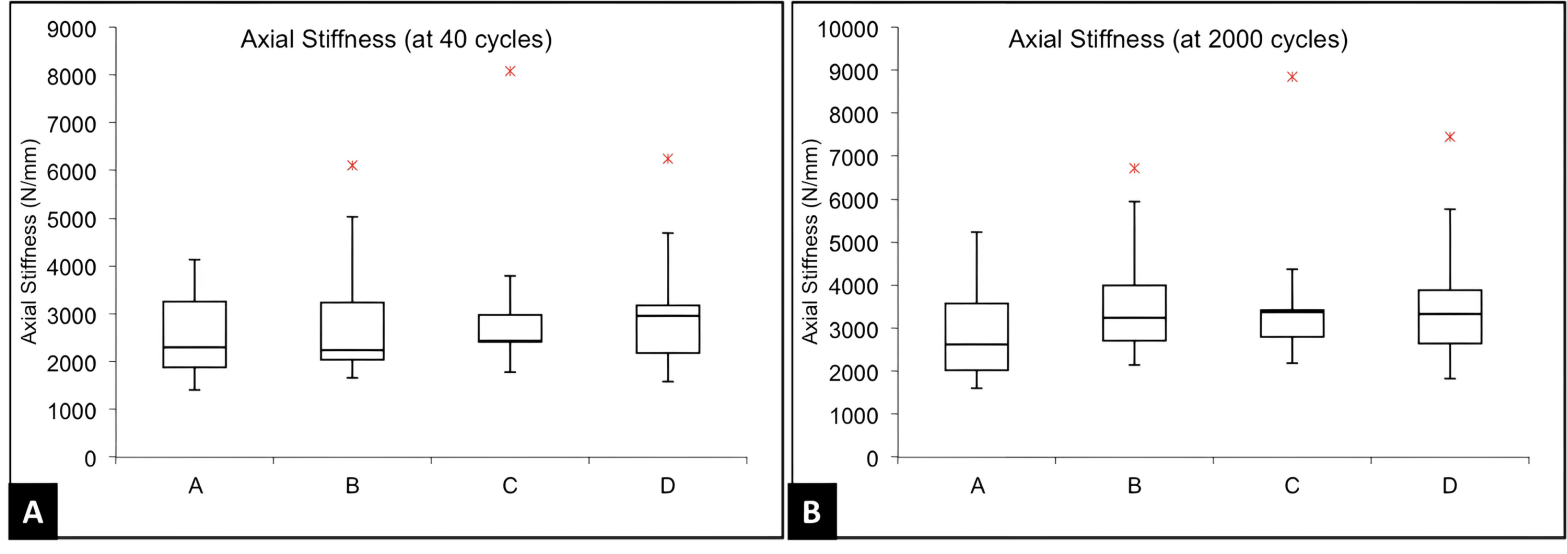
Comparison of axial stiffness at (A) 40 cycles and (B) 2000 cycles of compressive loading Box plot showing comparison of axial stiffness (N/mm) after 40 cycles and 2000 cycles of compressive loading across all four groups (A–D). In each boxplot, the horizontal line shows the median, the box represents the interquartile range (middle 50% of data), and the whiskers indicate the data spread within 1.5× interquartile range. Outliers are shown as individual dots. No statistically significant difference was observed among groups (p > 0.05).

The axial compressive stiffness at 40 cycles, 2000 cycles, and 4000 cycles was lowest for Group A and highest for Group D. However, one-way ANOVA did not reveal any significant differences in these parameters amongst the four study groups (Figure 7).

Construct failure and permanent deformation

No construct failure or permanent deformation was noted in any of the study groups after 4000 cycles of cyclical loading from 80 to 800 N.

Summary of results

All constructs completed mechanical testing without hardware failure. Group A, with the shortest distal fragment, consistently showed the least stability. In the AP plane, Group A had the lowest bending stiffness and the highest fracture gap angle and neutral zone, indicating more laxity. As the distal fragment length increased in groups B, C, and D, these parameters improved. Significant differences in AP bending stiffness and peak fracture gap angle were found between Group A and Group D (p < 0.05). In the ML plane, differences between groups were smaller and not statistically significant. Similarly, axial compressive stiffness showed no significant differences across groups at any loading cycle. Overall, shorter distal fragments mainly affect stability in the AP plane, while coronal and axial stability are less impacted.

## Discussion

The management of extra-articular distal tibial fractures continues to challenge orthopedic surgeons, particularly when the distal fragment is short. Although IM nailing remains the preferred method for its minimally invasive approach and biological preservation of the fracture environment, its mechanical stability can be compromised in the distal third of the tibia due to limited cortical contact and fewer distal locking options [25].

Our biomechanical study directly explored this concern by comparing construct stability across varying distal fragment lengths, simulating a spectrum of clinical scenarios. The findings clearly indicate that constructs with the shortest distal fragments (12% of tibial length) showed significantly reduced AP bending stiffness, increased neutral zone, and larger peak fracture gap angles compared to longer fragment constructs. This suggests that sagittal plane instability becomes more pronounced as the distal fragment shortens, a result that may not always be obvious intraoperatively but can have critical implications postoperatively [25,27].

Interestingly, while coronal plane instability often garners more clinical attention, this study underscores that sagittal plane control may be more vulnerable in short-fragment constructs. The increased neutral zone in the AP direction represents early toggle within the construct, a biomechanical behavior that translates clinically into delayed healing, malunion, or apex anterior deformity. This is particularly relevant in extra-articular fractures where small angular malalignments can have a disproportionate impact on functional outcomes, leading to altered ankle kinematics, anterior ankle impingement, and early degenerative changes [19,22].

Despite significant differences in AP stability, ML bending stiffness, and axial compressive stiffness were not significantly altered across groups. This implies that axial and coronal stability are relatively well-maintained by current IM nail designs and distal locking techniques. However, the greater sensitivity of the sagittal plane to fragment length indicates a need for targeted strategies to reinforce this axis, such as the addition of Poller (blocking) screws, multi-planar distal locking, or fibular fixation [22].

The clinical translation of these findings suggests caution when pursuing early ankle mobilization in cases with very short distal segments. Though early rehabilitation is often encouraged to minimize joint stiffness, it may increase the risk of loss of reduction in unstable constructs. Surgeons may consider modifying their approach either by augmenting stability intraoperatively or delaying certain phases of weight-bearing and physiotherapy postoperatively [22,25].

Our study also reaffirms the value of standardized, reproducible biomechanical testing. Fourth-generation composite tibial sawbones allowed consistent material properties across specimens, and testing focused on pure mechanical influence by eliminating biological variables. While this strengthens internal validity, several limitations are acknowledged. The sawbone model does not simulate bone healing or soft tissue forces; torsional forces, which are important in real-life gait, were not included due to equipment limitations. Additionally, no fibular fixation was employed, representing a worst-case scenario rather than standard practice [19,27].

Future research should explore whether sagittal instability can be mitigated through surgical technique modifications, including alternative nail designs with multi-planar distal locking, use of Poller screws, or hybrid constructs combining nails and plates. Clinical correlation studies are also essential to validate these findings and understand the thresholds of instability that predict poor outcomes [22].

## Conclusions

This biomechanical study highlights the critical impact of distal fragment length on construct stability following IM nailing in extra-articular distal tibial fractures. Our results show that when the distal fragment measures only 12% of the total tibial length, significant sagittal instability occurs, elevating the risk of malunion. Although IM nailing provides sufficient stability in the coronal and axial planes, its ability to control sagittal micromotion diminishes with reduced distal bone stock. These findings underscore the need to consider the 12% distal fragment length threshold during surgical planning to optimize fixation and minimize complications. Restricting early postoperative ankle motion may further reduce the risk of malalignment and improve outcomes.

This study also prompts an important question: Can augmentation techniques, such as Poller screws or angle-stable bolts, effectively address sagittal instability in short distal fragments, or do alternative fixation methods like locking plates offer better stability? Further research is needed to evaluate these strategies and guide surgical decision-making for improved patient recovery and long-term function.
